# The potential of visual evoked potentials latency and amplitude to be a subclinical predictor of clinical prognosis in multiple sclerosis

**DOI:** 10.1007/s11845-025-03980-x

**Published:** 2025-06-19

**Authors:** Elif Banu Soker, Miray Erdem, Derya Ozdogru, Onur Serdar Gencler, Onder Yesiloglu, Halit Fidanci

**Affiliations:** 1 Department of Neurology, Adana City Training and Research Hospital, Health Science University, Adana, Türkiye; 2 Department of Neurology, Ankara Bilkent City Hospital, Health Science University, Ankara, Türkiye; 3 Department of Emergency Medicine, 25 Aralik State Hospital, Gaziantep, Türkiye

**Keywords:** Multiple sclerosis, Optic neuritis, Remyelination, Short-term subclinical stability, Visual evoked potentials

## Abstract

**Background:**

Multiple sclerosis (MS) is a chronic inflammatory disease of the central nervous system characterized by focal demyelinating lesions, axonal dysfunction/degeneration, and gliosis, which can lead to various clinical disabilities. Visual evoked potentials (VEP) is sensitive and repeatable techniques capable of monitoring significant short-term changes in neuroaxonal integrity and alterations in nerve conduction triggered by acute optic neuritis.

**Aim:**

This study aims to evaluate whether VEP latency and amplitude could serve as subclinical predictors of clinical outcomes in MS patients over short-term follow-ups.

**Methods:**

This study was planned to include MS patients diagnosed according to the McDonald Criteria 18 who did not have any psychiatric, neurological, or ocular disorders that could interfere with the main purpose. The VEP test was performed for routine evaluation of demyelination or axonal damage.

**Results:**

A total of 83 patients were included in the study, with a mean age of 33.6 ± 9.3 years. Of all the patients, 54 were female (65.1%) and 29 were male (34.9%). Right pattern reversal visual evoked potential (PVEP) P100 (OR for PVEP1: 0.802, *p* = 0.001; OR for PVEP2: 0.879, *p* = 0.002) was statistically significant in showing right VEP abnormality at both baseline and at 6 months. Left VEP abnormalities were associated with left PVEP P100 at PVEP1 (OR: 0.852, *p* = 0.003) and left PVEP N75 at PVEP2 (OR: 0.935, *p* = 0.029).

**Conclusion:**

VEPs have the potential to predict short-term subclinical stability or progression, making them valuable candidates for early treatment adjustments and evaluating future pharmacotherapy-supported remyelination.

## Introduction

Multiple sclerosis (MS) is a chronic inflammatory disease of the central nervous system (CNS) characterized by focal demyelinating lesions, axonal dysfunction/degeneration, and gliosis, which can lead to various clinical disabilities. Myelinated axons are the primary target of MS attacks, and these episodes can cause varying degrees of damage to both the myelin and the axons themselves(1, 2). Currently, numerous treatment options exist for MS, and disease-modifying therapies (DMTs) have significant impacts on both disease activity and long-term disability(3). These results have been primarily achieved with anti-inflammatory drugs, as demonstrated by the reduction in emerging T2 lesions or gadolinium-enhancing lesions. Therefore, clinical efforts need to extend towards neuroprotection and remyelination to further expand the therapeutic options for MS(4). The repair of the myelin sheath is a therapeutic goal still not achieved in MS treatment and holds promise for improving functional recovery and preventing long-term disability, but our current treatments are still limited in terms of slowing disease progression(5–7).

Visual evoked potentials (VEP) and spectral domain optical coherence tomography (SD-OCT) are sensitive and repeatable techniques capable of monitoring significant short-term changes in neuroaxonal integrity and alterations in nerve conduction triggered by acute optic neuritis (AON)(8). VEP measures signal transmission along the entire visual pathway, and it is believed to primarily reflect the state of myelination(9–12). VEP is a reliable and relatively cheap method that can easily be utilized in clinical studies. The amplitude of the detected signal is assumed to be a parameter of axonal loss and reflects the number of functional fibers along the stimulated visual pathway. Increase in latency is a reliable in vivo marker of myelin repair(13).

The P100 latency is the main indicator of VEP, and it is a significant outcome candidate in studies on functional remyelination in MS. The literature shows that a Phase I randomized trial with liothyronine for remyelination in MS, as well as studies on patients treated with alemtuzumab, both with and without a history of optic neuritis, reported significant improvements in delayed P100 responses(14–17).

Previous studies have highlighted long-term follow-up results for alterations in treatment regimens. These studies have shown that VEP latency and amplitude are possible subclinical predictors during follow-ups between long intervals. However, literature data is limited regarding subclinical predictability based on VEP data obtained during a shorter period. Therefore, this study aims to evaluate whether VEP latency and amplitude could serve as subclinical predictors of clinical outcomes in MS patients over short-term follow-ups.

## Methods

Patients with multiple sclerosis (MS) who presented to the Neurology Clinic of Adana City Training and Research Hospital, affiliated with the Health Sciences University, between December 20, 2022, and February 20, 2023, were included in this retrospective cohort. The aim was to include all patients who met the inclusion criteria within the time period specified in the study design. Our study was planned to include MS patients diagnosed according to the McDonald Criteria(18) who did not have any psychiatric, neurological, or ocular disorders that could interfere with the main purpose. The VEP test was performed for routine evaluation of demyelination or axonal damage.

Pattern reversal visual evoked potentials (PVEPs) were performed on the patients at baseline (PVEP1) and 6 months afterward (PVEP2). Additionally, Expanded Disability Status Scale (EDSS) scores and visual acuity were assessed at baseline and after the 6th month(19). Ethical approval for the study was obtained from the local ethics committee (Health Science University, Adana City Research and Training Hospital Clinical Research Ethic Committee) (number: 118, description: 2320).

### Inclusion and exclusion criteria

Patients who had two tests at least 6 months apart were included in the study. Vascular or neurodegenerative comorbidities that could affect the optic nerve, additional medication use, use of more than one DMT within 6 months prior to the first VEP, use of more than one DMT for 6 months or longer between the two VEP examinations, not using any DMT for longer than 6 months, experiencing an episode during or within 3 months prior to the test, and any relapse diagnosed as optic neuritis at any time were determined as exclusion criteria for the study.

## Visual evoked potentials

The pattern visual evoked potential (PVEP) study was conducted using the Cadwell Sierra Summit Electromyography Unit (Cadwell Laboratories, Kennewick, Washington, USA) in a quiet, dark room. PVEP testing was performed with a CBOX 18.5″ LED monitor positioned 100 cm from the patient. A black-and-white checkerboard pattern with a red fixation dot at the center was used to guide the patient’s focus. Time interval between stimulus delivery and image display on the screen was 59 ms. This delay was corrected using the software integrated into the device. The checkerboard element size was 62 min of arc. The upper and lower frequency filters were set at 100 Hz and 1 Hz, respectively. According to the international 10–20 EEG system, a reference electrode was placed at Fz, and an active electrode was placed at Oz using silver cup electrodes. Impedance was maintained below 5 kΩ. The sweep speed was set at 25 ms/division, and the sensitivity was adjusted to 2.5–5 µV/division. The reference values from the clinical neurophysiology laboratory were used to determine the normal range for P100 wave latency(20). A P100 wave latency > 104.8 ms or a side-to-side difference > 10 ms was classified as abnormal. The signal from the mid-occipital electrode normally contains a prominent positive component that occurs approximately 100 ms after the stimulus and is called as P100. It is preceded by a smaller negative component called N75, which occurs at around 75 ms. The P100 latency obtained at the mid-electrode is accepted as a measure of retina-striatal conduction since waveforms at lateral electrodes are highly variable. Additionally, a P100 wave amplitude was considered abnormal if the wave could not be elicited or if the side-to-side difference exceeded 50%. All electrophysiological analyses were performed in our neurophysiology laboratory by a single neurophysiologist.

## Statistical analysis

Data obtained from the study were recorded using SPSS 28.0 (Armonk, NY: IBM Corp.) statistics program. Categorical measurements were recorded as numbers and percentages, while continuous measurements were recorded as mean and standard deviation. The normality of the distribution of continuous variables was evaluated with the Shapiro–Wilk test. Student’s *t*-test was used for parameters showing normal distribution and the Mann–Whitney *U* test was used for parameters not showing normal distribution while comparing between groups of continuous variables. The chi-square test or Fisher’s exact test was used for analysis of categorical variables. Multivariate regression analysis was conducted to determine the variables predicting VEP abnormalities at baseline and at 6 months, along with their levels of significance. The correlation between EDSS and visual acuity with VEP tests at baseline and at 6 months was evaluated with Spearman’s correlation test. A *p*-value of less than 0.05 was considered statistically significant.

## Results

A total of 83 patients were included in the study, with a mean age of 33.6 ± 9.3 years. Of all the patients, 54 were female (65.1%) and 29 were male (34.9%). A number of lesions in the cranial MRI T2 sequence, MS type, and demographic data of the patients are shown on Table 1. VEP (visual evoked potential) neuroelectrophysiological tests, EDSS, and visual acuity test were performed for all patients at baseline (PVEP1) and at the 6th month (PVEP2). The difference levels and statistical significance of these tests at these two time points are shown in Table 2. Visual acuity, measured on a scale of 10, showed a significant improvement at PVEP2 compared to PVEP1 (*p* = 0.029). According to the neuroelectrophysiological test results, right PVEP P100 (93.4 vs. 93.0, *p* = 0.015), left VEPn75 (57.8 vs. 56.6, *p* = 0.044), left PVEP P100 (95.1 vs. 94.1, *p* = 0.017), and left PVEP P100 amp levels at PVEP1 and PVEP2 (8.6 vs. 9.4, *p* < 0.001) were meaningfully different.

**Table 1:** Clinical characteristics of the study population

Variable	*n* = 83
Age, years (± s.d.)	33.6 ± 9.3
Sex, female, *n* (%)	54 (65.1)
Multiple sclerosis type, *n* (%) *Relapsing–remitting secondary progressive*	70 (84.3) 13 (15.7)
Cranial magnetic resonance imaging, *n* (%) *0–20* *20–50* *50–100*	31 (37.3) 38 (45.8) 14 (16.9)
Spinal magnetic resonance imaging, *n* (%) *None* *0–10* > *10*	31 (37.3) 44 (53.0) 8 (9.6)
Medical treatments, *n* (%) *Injections* *Teriflunamide* *Dimethyl fumarate* *Fingolimod* *Ocreus* *Natalizumab*	19 (22.9) 13 (15.7) 18 (21.7) 15 (18.1) 10 (12.0) 8 (9.6)

**Table 2:** Differences and significance levels of EDSS, visual acuity, and PVEP values at 6th month compared to baseline

	Baseline	6th month	*p* value
EDSS, *m* (IQR)	1.00 (1.0)	1.00 (1.0)	0.788
Visual acuity, out of 10 (mean ± s.d.)	9.5 ± 0.8	9.7 ± 0.5	0.029
Right PVEPN75, *m* (IQR)	62.10 (14.8)	60.50 (16.2)	0.392
Right PVEPP100, *m* (IQR)	93.40 (19.0)	93.00 (17.6)	0.015
Right PVEPN145, *m* (IQR)	140.50 (19.9)	139.80 (18.7)	0.204
Right PVEPP100amp (mean ± s.d.)	8.65 ± 4.7	9.36 ± 4.5	0.057
Left PVEPN75, *m* (IQR)	57.8 (16.6)	56.6 (17.9)	0.044
Left PVEPP100, *m* (IQR)	95.1 (16.8)	94.1 (20.0)	0.017
Left PVEPN145, *m* (IQR)	135.7 (22.5)	133.5 (19.9)	0.528
Left PVEPP100amp, (mean ± s.d.)	8.6 ± 4.9	9.4 ± 5.0	< 0.001
*Right PVEP abnormality, *n* (%)	30 (36.1)	24 (28.9)	0.146
*Left PVEP abnormality	26 (31.3)	27 (32.5)	1.000
*Right or left PVEP abnormality	37 (44.6)	31 (37.3)	0.180

^*^McNemar test (*EDSS* Expanded Disability Status Scale, *PVEP* pattern visual evoked potential)

The correlation between EDSS and visual acuity with VEP neuroelectrophysiological values was examined in Table 3 as PVEP1 and PVEP2. Right PVEP N75 (EDSS PVEP1 *p* = 0.027, visual acuity PVEP1 *p* = 0.001, EDSS PVEP2 *p* = 0.002, visual acuity PVEP2 *p* = 0.022), right PVEP p100 (EDSS PVEP1 *p* = 0.007, visual acuity PVEP1 *p* < 0.001, EDSS PVEP2 *p* < 0.001, visual acuity PVEP2 *p* < 0.001), and left PVEP P100 (EDSS PVEP1 *p* = 0.002, visual acuity PVEP1 *p* < 0.001, EDSS PVEP2 *p* < 0.001, visual acuity PVEP2 *p* < 0.001) tests showed a significant positive correlation with both clinical evaluation criteria at baseline and at 6 months. The results of multivariate logistic regression models used to predict VEP abnormalities at baseline and at 6 months are presented in Table 4. According to this model based on neuroelectrophysiological VEP tests, right PVEP P100 (OR for PVEP1: 0.802, *p* = 0.001; OR for PVEP2: 0.879, *p* = 0.002) was statistically significant in showing right VEP abnormality at both baseline and at 6 months. Left VEP abnormalities were associated with left PVEP P100 at PVEP1 (OR: 0.852, *p* = 0.003) and left PVEP N75 at PVEP2 (OR: 0.935, *p* = 0.029).

**Table 3:** Correlation values of EDSS and visual acuity with PVEP tests at baseline and 6th periods

	Baseline				6th month			
	EDSS		Visual acuity		EDSS		Visual acuity	
	Rho	*p*	Rho	*p*	Rho	*p*	Rho	*p*
Right PVEPN75	0.255	0.027	0.374	0.001	0.350	0.002	0.258	0.022
Right PVEPP100	0.309	0.007	0.441	< 0.001	0.413	< 0.001	0.453	< 0.001
Right PVEPN145	0.166	0.155	0.265	0.022	0.312	0.005	0.191	0.092
Right PVEPP100 amp	− 0.31	0.779	− 0.205	0.063	− 0.110	0.323	− 0.197	0.074
Left PVEPN75	0.213	0.063	0.392	< 0.001	0.415	< 0.001	0.401	< 0.001
Left PVEPP100	0.355	0.002	0.434	< 0.001	0.483	< 0.001	0.486	< 0.001
Left PVEPN145	0.171	0.137	0.281	0.013	0.275	0.016	0.363	0.001
Left PVEPP100 amp	− 0.113	0.307	− 0.351	0.001	− 0.091	0.416	− 0.182	0.099

*EDSS* Expanded Disability Status Scale, *PVEP*: pattern visual evoked potential, *amp* amplitude

**Table 4:** Multivariate logistic regression analysis for PVEP abnormality

	Right PVEP abnormality			
	Baseline		6th month	
Variable	OR (95% CI)	*p* value	OR (95% CI)	*p* value
Right PVEPN75	0.941 (0.828–1.069)	0.352	1.011 (0.944–1.082)	0.352
Right PVEPP100	0.802 (0.701–0.916)	0.001	0.879 (0.811–0.953)	0.002
Right PVEPN145	1.055 (0.956–1.056)	0.854	1.008 (0.956–1.056)	0.709
Right PVEPP100 amp	0.955 (0.716–1.276)	0.757	1.122 (0.912–1.382)	0.276
	Left PVEP abnormality			
	Baseline		6th month	
	OR (95% CI)	*p* value	OR (95% CI)	*p* value
Left PVEPN75	1.011 (0.947–1.079)	0.747	0.935 (0.880–0.993)	0.029
Left PVEPP100	0.852 (0.766–0.947)	0.003	0.955 (0.883–1.033)	0.254
Left PVEPN145	1.055 (0.988–1.124)	0.110	1.033 (0.975–1.094)	0.272
Left PVEPP100 amp	1.087 (0.929–1.272)	0.298	1.065 (0.932–1.218)	0.355

*EDSS* Expanded Disability Status Scale, *PVEP* pattern visual evoked potential, *amp* amplitude

## Discussion

This study analyzes short-term changes in VEP, EDSS, and visual acuity in MS patients, highlighting the correlations between these parameters. Our findings suggest that VEP parameters in particular are important indicators in demonstrating short-term clinical improvement. In our study, visual acuity showed a significant improvement at the sixth month compared to the initial presentation. Among the neuroelectrophysiological tests, meaningful differences were found between PVEP1 and PVEP2 in right PVEP P100, left VEP N75, left PVEP P100, and left PVEP P100 amplitude levels. Accordingly, significant positive correlations were found between mentioned clinical evaluation measures and the right PVEP N75, right PVEP P100, and left PVEP P100 tests, both at the initial visit and during the 6-month follow-up.

Based on the literature, identifying the factors that render the cerebral hemispheres more susceptible to autoimmune damage appears to be challenging. Some authors attribute this asymmetrical distribution of lesions to left hemispheres greater susceptibility to neural and metabolic dysfunction(21). Another study states that their findings suggested VEP score of the left eye had predictive values in achieving NEDA-3 (no evidence of disease activity) over 10 years in MS patients receiving first-line immunomodulatory treatment. These findings could explain the greater likelihood of changes in the left optic nerve(22).

MS is a disease characterized by demyelination of neurons in the central nervous system, and it can cause visual impairment by particularly affecting the optic nerves rich in myelin(23). While VEP is not among the diagnostic criteria for MS(24), it can be utilized during the follow-up of clinical improvement in visual function. MRI has admittedly transformed MS diagnosis, but VEPs offer a modest increase in the sensitivity of modern diagnostic criteria by incorporating additional regions, such as the optic nerve, into the dissemination in space criteria (DIS)(25). Despite the absence of VEPs in currently published modern diagnostic criteria for MS, some studies similar to ours have shown the utility of electrophysiological monitoring to evaluate disease progression. Longitudinal VEP measurements in relapsing MS patients treated with β-interferon and fingolimod, along with prognostic modeling by Schlaeger et al.(26), which combines VEP and motor evoked potentials to predict future disability up to 20 years, are part of the recent advancements in this field(26–28). These studies suggest that long-term treatment response is being researched on and VEP measurements can be effective in predicting clinical improvement. In our study, we found significant changes in right and left PVEP P100 and N75 latencies at the end of the 6-month follow-up period. This notable improvement in right PVEP P100 latency and the change in left PVEP P100 amplitude indicate that treatments lead to enhanced optic nerve conduction in a relatively short period. When evaluated together with the significant improvement in visual acuity observed during the follow-up period, it supports the validity of our VEP results. The earlier observation of treatment response also leads us to believe that VEP parameters are quite valuable in showing subclinical progression during the asymptomatic period in cases where the disease involves the optic nerve.

In our study, a significant correlation was found between EDSS and visual acuity with VEP neuroelectrophysiological values. A positive relationship was found between right PVEP p100 and left PVEP P100 tests and both clinical evaluation criteria at baseline and at 6 months. According to literature findings, VEPs correlate more weakly with EDSS, a common clinical measure of MS disability, compared to motor or sensory evoked potentials(29). However, our results show that the presence of a strong relationship between VEP parameters and EDSS suggests VEP has the potential not only to evaluate visual function but also to reflect the disability of the disease. When EDSS, visual acuity, and VEP analyses are evaluated together, it effectively highlights that VEP is an important tool in understanding clinical progression in MS patients.

The multivariate logistic regression analyses in our study indicate that right PVEP P100 values are particularly reliable predictors of VEP abnormalities at both baseline and the 6-month follow-up. A study evaluated the sensitivity of VEP measurements in detecting optic neuritis in MS patients emphasizes that VEP measurements can be a useful tool in showing abnormality, especially in patients without optic neuritis^30^. The multivariate logistic regression analysis in our study shows that VEP measurements can serve as an objective marker for planning follow-up in MS patients.

## Limitations of the study

This study also has some limitations. The limited sample size, primarily due to the study being conducted at a single center, is a key limitation of our study. Another limitation of our study is the difficulty in establishing a clear relationship between the medications used by the study participants and VEP parameters. This limitation is likely due to the inability to perform subgroup analyses based on the medications used, given the insufficient number of patients. The last limitation is that the sample calculation could not be done to reveal the power of the study. Since our patient number is a small disease, the maximum number of patients who met the inclusion criteria were included in the time period we determined.

## Conclusion

VEPs have the potential to predict short-term subclinical stability or progression, making them valuable candidates for early treatment adjustments and evaluating future pharmacotherapy-supported remyelination. Visual neuroelectrophysiology remains a crucial tool for clinicians in both clinical practice and assessing treatment responses.

## Data Availability

Data and materials are reachable from hospital automation information systems.
